# Prescription Sequence Symmetry Analysis of Glucagon‐Like Peptide‐1 Receptor Agonists and Neuropsychiatric Conditions

**DOI:** 10.1002/cpt.70414

**Published:** 2026-07-30

**Authors:** Maria J. Alfonso Arvez, Sam Wade, Darshna Goordeen, Jenni Ilomäki, Amanda J. Cross, Monica Langiu, George S. Q. Tan

**Affiliations:** ^1^ Centre for Medicine Use and Safety, Faculty of Pharmacy and Pharmaceutical Sciences Monash University Parkville Victoria Australia; ^2^ Drug Discovery Biology, Monash Institute of Pharmaceutical Sciences, Faculty of Pharmacy and Pharmaceutical Sciences Monash University Parkville Victoria Australia; ^3^ AK Clinical Research Carlton Victoria Australia

## Abstract

Glucagon‐like peptide‐1 receptor agonists (GLP‐1RAs) are increasingly prescribed for type 2 diabetes and weight management. However, conflicting evidence from preclinical, clinical, and pharmacovigilance studies suggests potential neuropsychiatric effects. This study examined associations between GLP‐1RA use and initiation of a range of neuropsychiatric medications using real‐world prescription data. A Prescription Sequence Symmetry Analysis was conducted using Australia's Pharmaceutical Benefits Scheme 10% random sample between July 1, 2013 and December 31, 2024. Individuals with an incident dispensing of both a GLP‐1RA and a neuropsychiatric medication were included. Outcomes comprised antidepressants, medication for substance use disorder (SUD), antipsychotics, psychostimulants, antidementia medications, antiparkinsonian medications, antiepileptics, and antimigraine agents. Adjusted sequence ratios (aSRs) with 95% confidence intervals (CIs) were calculated using a one‐year exposure window, with sensitivity analyses varying various time windows. Among 2,033 individuals, semaglutide was the most common GLP‐1RA (50.6%), followed by exenatide (27.5%) and dulaglutide (21.9%). GLP‐1RA initiation was inversely associated with initiation of antidepressants (aSR: 0.85; 95% CI: 0.76–0.94) and medications for SUD (aSR: 0.70; 95% CI: 0.51–0.88). No significant associations were observed for other neuropsychiatric outcomes. GLP‐1RA use was inversely associated with subsequent initiation of antidepressants and medications for SUD, while no associations were identified for other neuropsychiatric marker medications. These findings highlight the need for further studies to clarify the nature and magnitude of these potential neuropsychiatric associations with GLP‐1RAs.

**Study Highlights**

**WHAT IS THE CURRENT KNOWLEDGE ON THE TOPIC?**

Glucagon‐like peptide‐1 receptor agonists (GLP‐1RAs) are a class of glucose‐lowering medications that have additionally been repurposed for weight management. There is an increasing interest in the potential neuropsychiatric effects of GLP‐1RAs, with conflicting potential therapeutic benefits reported across mood disorders, substance use disorders, and neurocognitive conditions.

**WHAT QUESTION DID THIS STUDY ADDRESS?**

Is the use of GLP‐1RAs associated with neuropsychiatric conditions in real‐world clinical settings?

**WHAT DOES THIS STUDY ADD TO OUR KNOWLEDGE?**

In this nationwide prescription sequence symmetry analysis in Australia, GLP‐1RA initiation was inversely associated with subsequent initiation of antidepressants and medications for substance use disorders. No significant associations were observed for antipsychotics, psychostimulants, antidementia, antiparkinsonian, antiepileptics, or antimigraine agents.

**HOW MIGHT THIS CHANGE CLINICAL PHARMACOLOGY OR TRANSLATIONAL SCIENCE?**

GLP‐1RA use resulted in an inverse association with subsequent initiation of medications used for depression and substance use disorders, supporting the need to conduct further research into their potential neuropsychiatric effects and opportunity for drug repurposing.

Glucagon‐like peptide‐1 (GLP‐1) receptor agonists (GLP‐1RAs) mimic the endogenous incretin hormone GLP‐1 to enhance glucose‐dependent insulin secretion and suppress glucagon release. Originally indicated for the treatment of type 2 diabetes mellitus, GLP‐1RAs have now been approved for weight management and have demonstrated cardiorenoprotective benefits.^1^ Examples of GLP‐1RAs include exenatide, liraglutide, dulaglutide, and semaglutide. GLP‐1RAs are currently recommended by the American Diabetes Association guidelines as a glucose‐lowering add‐on therapy in people with atherosclerotic cardiovascular disease or chronic kidney disease.^2^, ^3^ These new indications and additional benefits of GLP‐1RAs have led to a rapidly expanding use of GLP‐1RAs globally.^4^, ^5^

In recent years, there has been a growing body of evidence on the potential neuropsychiatric effects of GLP‐1RAs. Preclinical studies have investigated the potential effects of GLP‐1RAs on the central nervous system and shown that GLP‐1RAs can decrease oxidative stress and inflammatory responses, as well as modulate neuronal pathways.^1^, ^6^ These neuroprotective and neuromodulatory properties have sparked significant interest in exploring the use of GLP‐1RAs to treat neurological, neurodegenerative, and mental health disorders.

The widespread use of GLP‐1RAs has also led to an increase in reports of psychiatric adverse events. Analysis of individual case safety reports from the World Health Organization's and United States Food and Drug Administration's pharmacovigilance databases showed disproportionately higher reporting of nervousness, agitation, depression, insomnia, and suicidal ideation.^7^, ^8^, ^9^ However, a large systematic review and meta‐analysis of 80 randomized controlled trials (RCTs) has found no evidence of increasing risk of both serious and nonserious psychiatric adverse events following GLP‐1RA use.^10^ While existing preclinical and clinical evidence may be conflicting, analysis of the increasing mass of real‐world data capturing GLP‐1RA use and clinical outcomes may provide further insight into whether GLP‐1RA use confers neuropsychiatric benefits or harms.

In this study, we performed an exploratory signal‐detection analysis on a large nationwide prescription claims database. Using a prescription sequence symmetry analysis, we examined the association between GLP‐1RA use and subsequent initiation of a wide range of neuropsychiatric medications.

## MATERIALS AND METHODS

### Data source

The Australian Pharmaceutical Benefits Scheme (PBS) is a national single‐payer scheme to provide pharmaceutical coverage to Australian citizens, permanent residents, and foreign visitors via reciprocal healthcare agreements.^11^ The PBS subsidizes approximately 75% of prescribed medications in Australia.^11^

We used the 10% sample of PBS data, which consists of nationwide PBS‐subsidized prescription claims records from community pharmacies, private hospitals, and hospital discharge from public hospitals for a random 10% sample of PBS‐eligible people living in Australia.^11^ The data include basic demographic information (sex, year of birth, year of death) and prescription claim information (PBS item code, date of prescribing and dispensing, and quantity supplied). For those with a recorded year of death, the date of death was estimated to be 2 months after their last dispensing record. PBS item codes were mapped to the World Health Organization's Anatomical Therapeutic Chemical (ATC) codes.^12^

### Study design

We used a Prescription Sequence Symmetry Analysis (PSSA) approach to evaluate the association between GLP‐1RA use and a wide range of neuropsychiatric medications. The PSSA design is a well‐established signal detection method used to assess suspected drug safety signals.^13^, ^14^, ^15^ It was later adapted as a hypothesis‐free screening method to identify unsuspected drug‐outcome associations for drug safety signal detection,^16^ and more recently, drug repurposing signal generation.^17^ As a cohort crossover design, it is analogous to a self‐controlled design and controls for time‐invariant individual‐level confounders such as sex, genetic or environmental factors.^18^

PSSA estimates the prescription sequence ratio (SR), which compares the number of people who initiate the exposure medication of interest (i.e., index medication) before the medication that is a proxy for the outcome condition of interest (i.e., marker medication), to those who initiate it after (i.e., marker medication then index medication).^13^, ^14^ Assuming no association between the index and marker medication, we would expect a symmetrical distribution of people initiating either the index or marker medication first. If use of the index medication is positively associated with the outcome, we would observe an excess of people initiating the index medication first. On the other hand, if use of the index medication is inversely associated with the outcome, we would observe an excess of people initiating the marker medication first.

GLP‐1RA was defined as the index medication. During the study period, GLP‐1RAs were subsidized under the PBS for the treatment of type 2 diabetes and not for weight management. We evaluated the association between GLP‐1RA use and eight groups of marker medications, each serving as a proxy for a corresponding neuropsychiatric condition: antidepressants (ATC codes start with N06A; depression), medications for substance use disorders (N07B; substance use disorder, SUD), antipsychotics (N05A; schizophrenia), psychostimulants (N06B; attention deficit hyperactivity disorder, ADHD), antidementia medications (N06D; Alzheimer's disease), antiparkinsonian medications (N04; Parkinson's disease), antiepileptics (N03A; epilepsy), and antimigraine agents (N02C; migraine). Medications for SUD subsidized under the PBS included medications for alcohol use disorder, tobacco use disorder, and opioid use disorder. Antidementia medications are only subsidized under the PBS for Alzheimer's disease and not for other forms of dementia. We only include antimigraine agents specifically indicated for acute migraine or migraine prevention, including Calcitonin Gene‐Related Peptide (CGRP) ‐targeting monoclonal antibodies. The full list of index and marker medications, alongside their corresponding ATC and PBS item codes, is listed in **Table** **S1**.

### Study cohort

**Figure 1:**
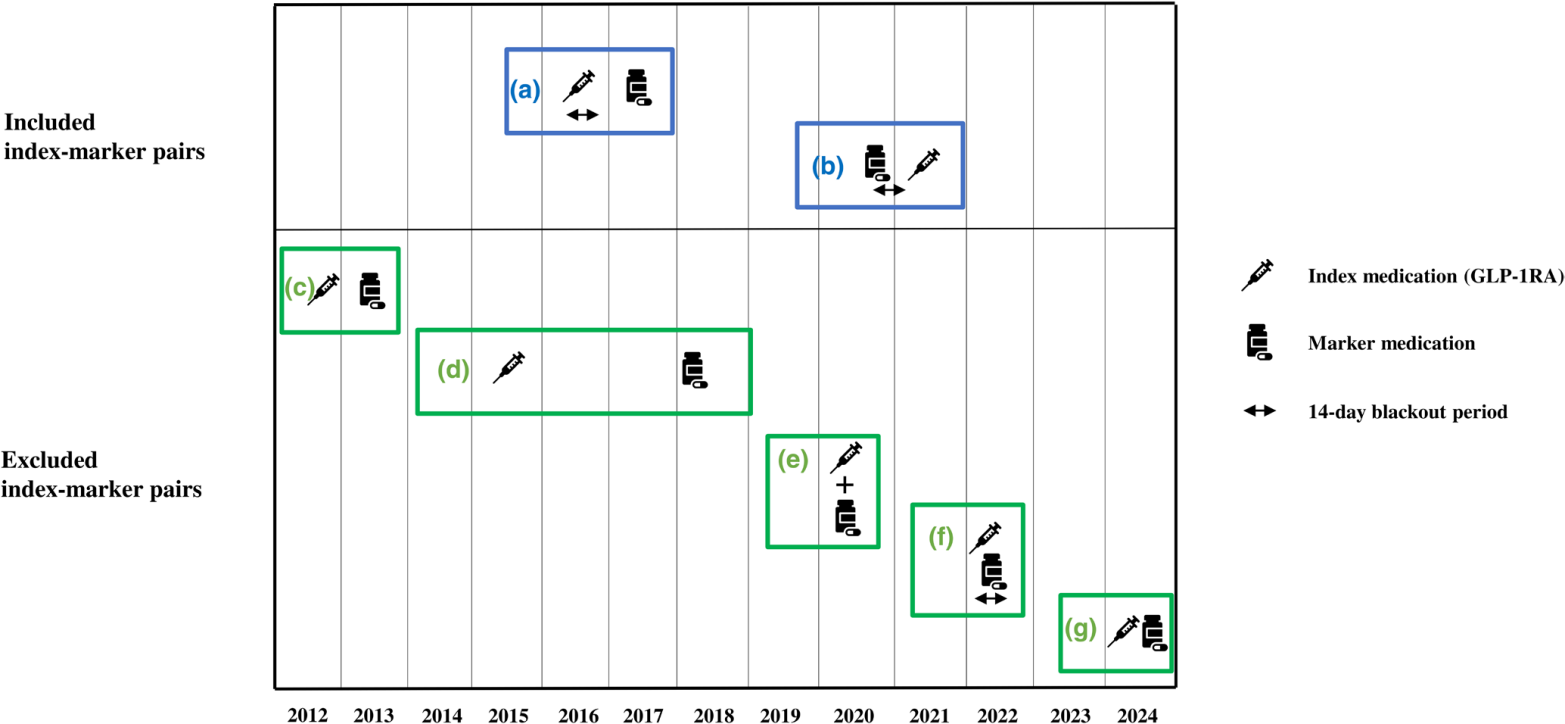
Sequence symmetry analysis study design with examples of included (A–B) and excluded (C–G) index‐marker medication pairs. A: index followed by marker medication; B: marker followed by index medication; C: excluded since < 1 year washout window to define incident use of index medication; D: excluded since index and marker medication > 1‐year exposure window apart; E: excluded since index and marker medication initiated on the same day; F: excluded since index and marker medication < 14‐day blackout period; G: excluded since < 1‐year follow‐up period. Adapted from Hendrix *et al*.^20^

People were included in this study if they were dispensed at least one GLP‐1RA and one marker medication between July 1, 2013 and December 31, 2024. We only included the first‐ever incident dispensing of GLP‐1RA and marker medications, using a 1‐year washout window. A one‐year exposure window was used to capture pairs of incident GLP‐1RA and marker medication use by the same individual, i.e., GLP‐1RA and the marker medication were initiated within 365 days of each other (**Figure** **1**). A one‐year exposure window has been shown to offer better specificity and positive predictive value compared to shorter windows.^19^ Accordingly, we limited the primary analysis to a one‐year exposure window to minimize the influence of aging and other time‐varying covariates. People were excluded if they initiated GLP‐1RA and the marker medication on the same day. People with exposure windows shorter than 1 year, e.g., people who initiated both index and marker medications within 1 year prior to the end of the data period (December 31, 2024) or recorded death, were also excluded. To minimize spurious associations due to, e.g., reverse causation, we used a 14‐day blackout window to exclude people who initiated both GLP‐1RA and the marker medications within a 14‐day period.^20^

### Statistical analyses

The crude sequence ratio (cSR) was estimated using previously described methods.^13^ As the cSR is sensitive to underlying trends of medication use over time,^18^ adjustment for temporal trends was performed using the null‐effect SR, as described by Tsiropoulos *et al*.^21^ An adjusted sequence ratio (aSR) was estimated by dividing the cSR by the null‐effect SR. Confidence intervals (CI) for the aSRs were calculated as described previously.^20^ An aSR with a 95% CI lower bound limit above 1 suggests a positive association, whereas an aSR with a 95% CI upper bound limit below 1 suggests an inverse association.

Sensitivity analyses were conducted to assess the robustness of the findings. First, the one‐year exposure window was extended to 2 years to account for potential delayed or cumulative effects of the index medication and outcomes with long latency due to diagnostic delays, such as Alzheimer's disease. Second, the 14‐day blackout window was extended to 6 months to reduce the risk of detecting spurious associations for outcomes with long latency. Third, the 1‐year washout window was extended to 2 years to reduce the risk of misclassifying relapsing psychiatric conditions, such as depression, as incident cases.

All statistical analyses were conducted using SAS software version 9.4 (SAS Institute Inc., Cary, North Carolina), and R version 4.4.2.

### Reporting guideline

This study is reported in accordance with the reporting of studies conducted using observational routinely‐collected health data (RECORD) guidelines.^22^ The completed RECORD checklist is provided in **Table** **S2**.

### Ethics Statement

This study was approved by the Monash University Human Research Ethics Committee (22877). The analysis application was approved and the manuscript noted by Services Australia's External Request Evaluation Committee (RMS4443).

## RESULTS

**Figure 2:**
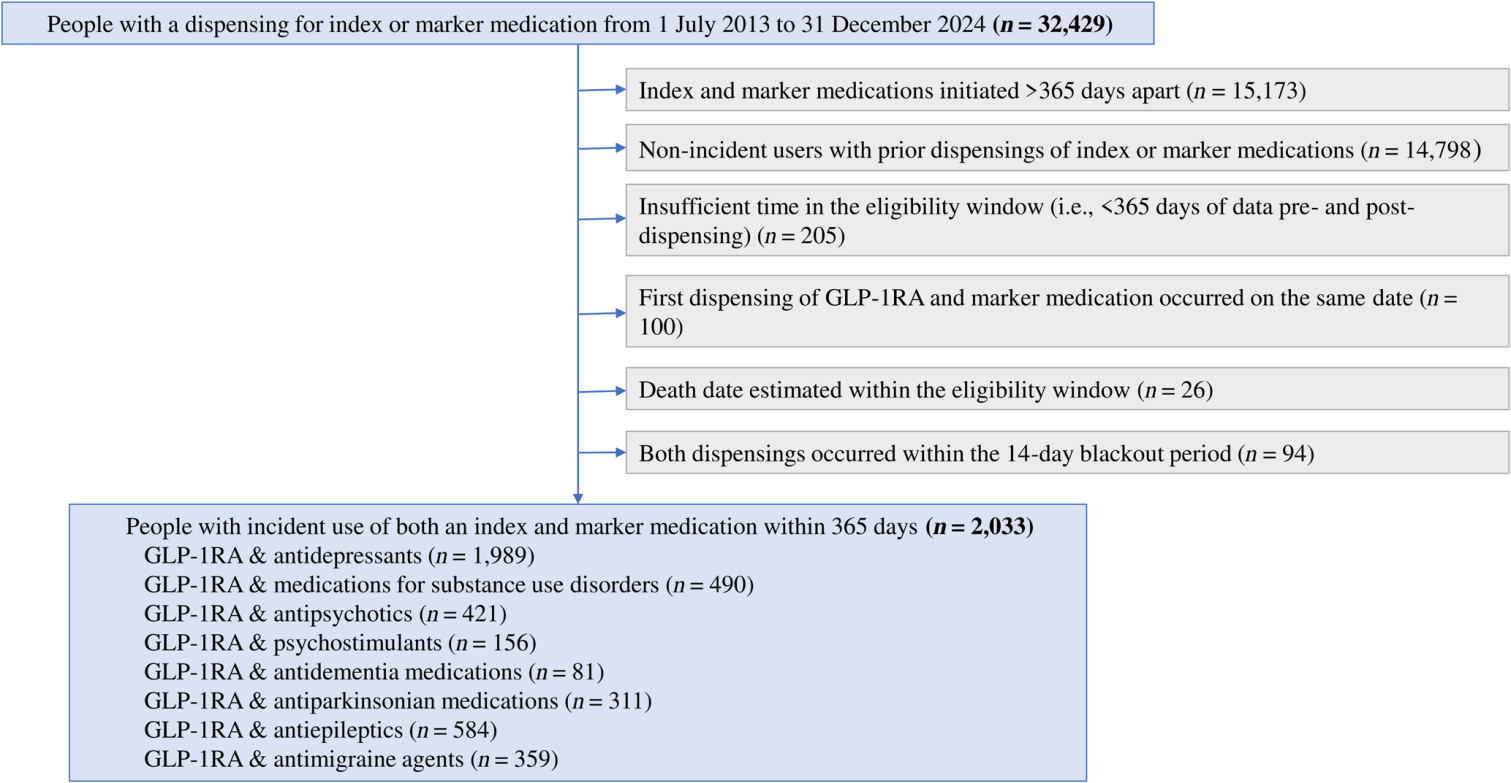
Flowchart for obtaining the cohort. Medication groups are not mutually exclusive, and an individual with multiple eligible marker dispensings will contribute to multiple groups. GLP‐1RA, glucagon‐like peptide‐1 receptor agonist. These are just bolded to highlight the start and the end sizes of the cohort.

A total of 32,429 individuals were dispensed at least one GLP‐1RA and one marker medication between July 1, 2013 and December 31, 2024. After exclusion criteria were applied, the final analytical cohort included 2,033 individuals (**Figure** **2**). Approximately half of the cohort were male (1,069; 52.6%); the median age of the cohort was 59 years (IQR: 49–69). Semaglutide was the most common GLP‐1RA initiated (1,028; 50.6%), followed by exenatide (559; 27.5%) and dulaglutide (446; 21.9%). Most of the cohort initiated GLP‐1RA between 2021 and 2023 (**Table** **1**).

**Table 1:** Baseline characteristics of identified cohort

	Identified Cohort
Total, No. (%)	2,033 (100%)
Age (years) at GLP‐1RA Initiation	
Median (IQR)	59 (49 to 69)
Age groups, No. (%)	
0–17	11 (0.5%)
18–29	69 (3.4%)
30–39	161 (7.9%)
40–49	297 (14.6%)
50–59	515 (25.3%)
60–69	515 (25.3%)
70–79	363 (17.9%)
80+	102 (5.0%)
Sex, No. (%)	
Male	1,069 (52.6%)
Female	964 (47.4%)
Index GLP‐1RA, No. (%)	
Exenatide	559 (27.5%)
Dulaglutide	446 (21.9%)
Semaglutide	1,028 (50.6%)
Year of Index, No. (%)	
2013	9 (0.4%)
2014	60 (3.0%)
2015	81 (4.0%)
2016	118 (5.8%)
2017	123 (6.1%)
2018	114 (5.6%)
2019	157 (7.7%)
2020	141 (6.9%)
2021	344 (16.9%)
2022	372 (18.3%)
2023	415 (20.4%)
2024	99 (4.9%)

GLP‐1RA, glucagon‐like peptide‐1 receptor agonists; IQR, interquartile range.

**Figure 3:**
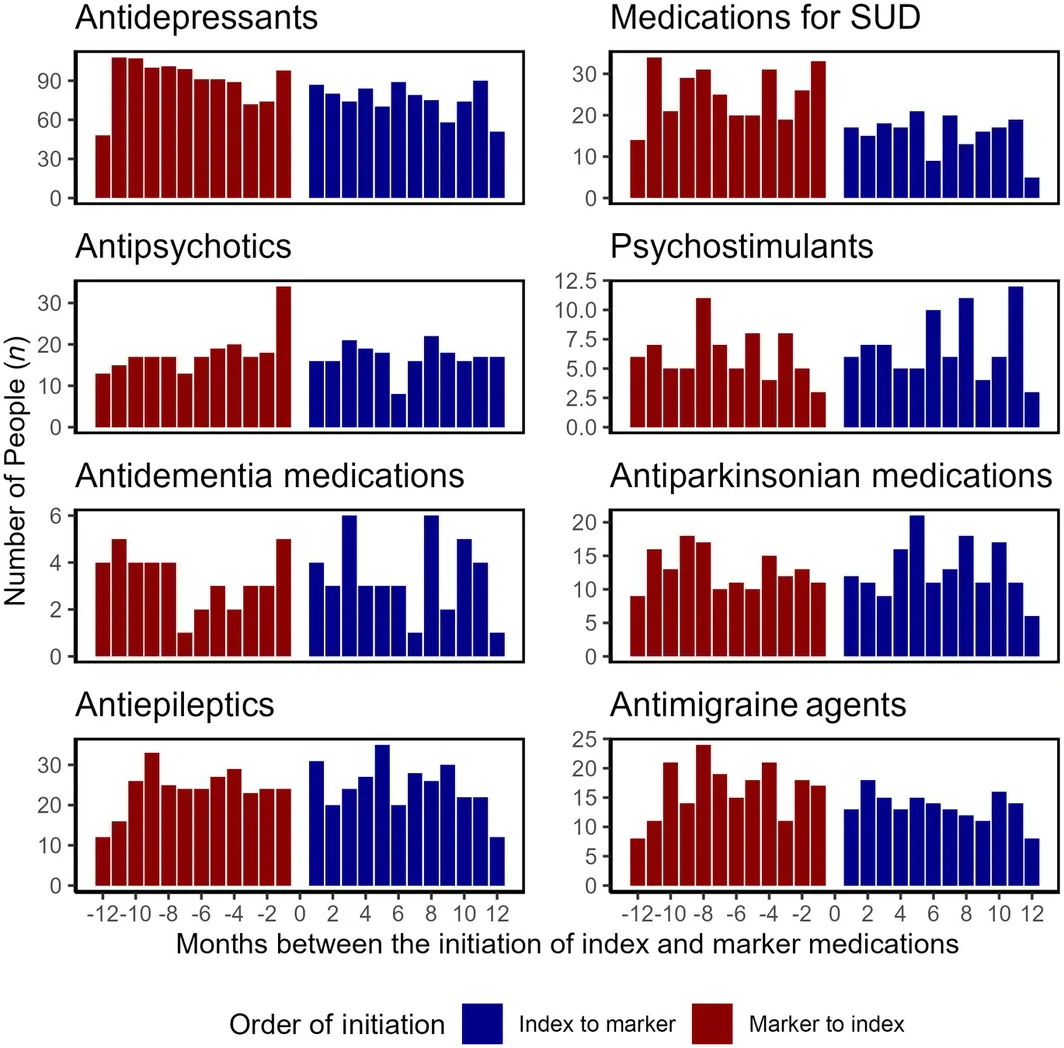
Sequence symmetry analyses for incident GLP‐1RA and marker medication dispensing.

PSSA identified inverse associations between GLP‐1RA and antidepressants (aSR: 0.85, 95% CI: 0.76–0.94) and medications for SUD (aSR: 0.70, 95% CI: 0.51–0.88). No significant associations were observed for other marker medications, including antipsychotics (aSR: 0.90, 95% CI: 0.71–1.09), psychostimulants (aSR: 1.01, 95% CI: 0.69–1.32), antidementia medications (aSR: 0.91, 95% CI: 0.47–1.34), antiparkinsonian medications (aSR: 0.94, 95% CI: 0.72–1.17), antiepileptics (aSR: 1.04, 95% CI: 0.88–1.20), and antimigraine agents (aSR: 0.84, 95% CI: 0.64–1.05) (**Figures** **3** **and** **4**).

**Figure 4:**
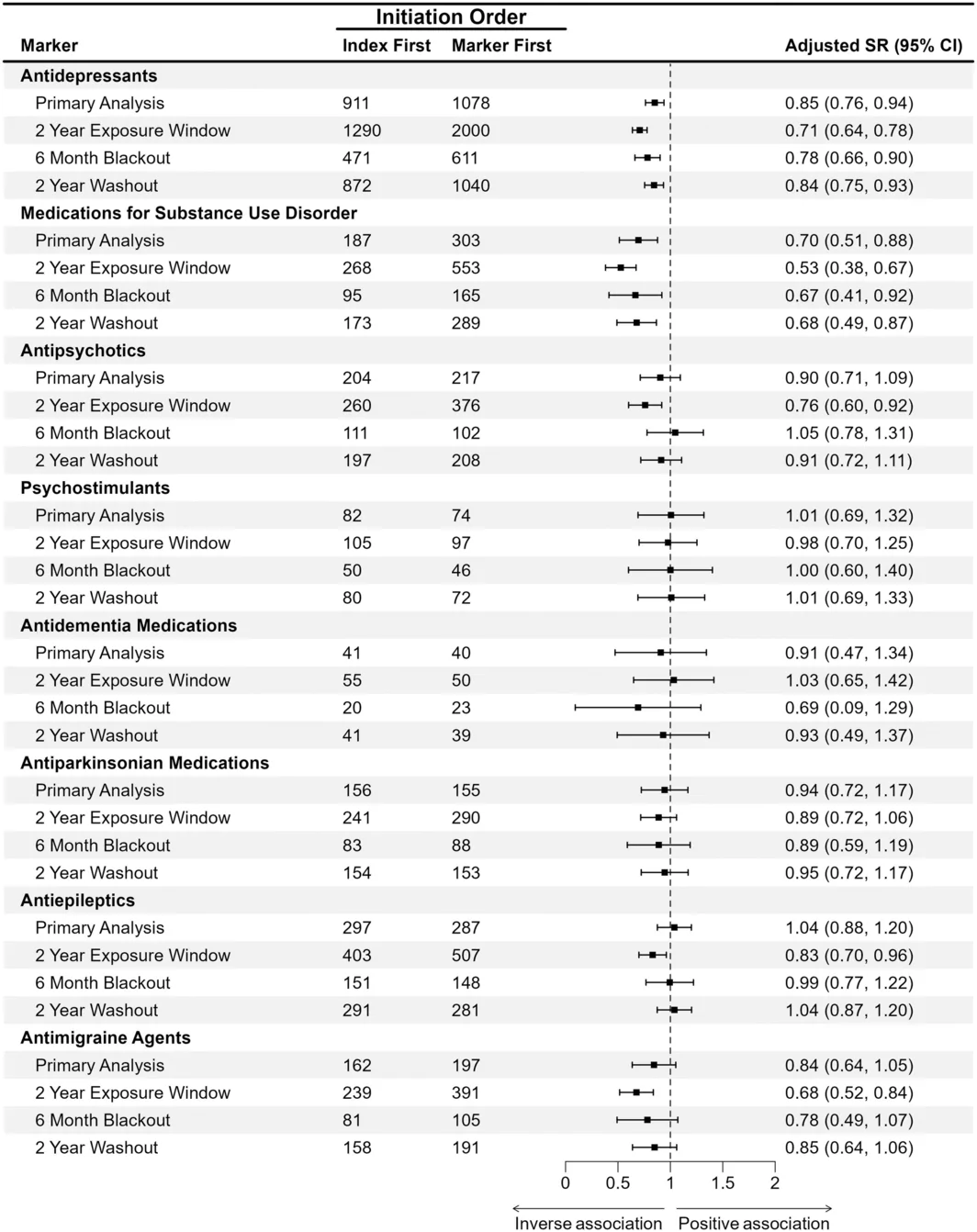
Forest plot showing adjusted sequence ratios (aSRs) for GLP‐1RA and marker medication dispensing in primary and sensitivity analyses.

The sensitivity analyses involving a two‐year exposure window found empirically larger inverse associations for antidepressants (aSR: 0.71, 95% CI: 0.64–0.78) and medications for SUD (aSR: 0.53, 95% CI: 0.38–0.67) (**Figure** **4**). When using the longer two‐year exposure window, inverse associations were also observed for antipsychotics (aSR: 0.76, 95% CI: 0.60–0.92), antiepileptics (aSR: 0.83, 95% CI: 0.70–0.96), and antimigraine agents (aSR: 0.68, 95% CI: 0.52–0.84). Results for the other sensitivity analyses involving longer blackout window and longer washout window remained consistent with those from the primary analyses (**Figure** **4**).

## DISCUSSION

This is the first study to examine the association between GLP‐1RA use and a wide range of medications for neuropsychiatric conditions, using a PSSA study design. We observed inverse associations between initiating GLP‐1RAs and antidepressants as well as medications for SUD, and these associations were more pronounced in the longer two‐year window sensitivity analysis. While this study did not identify any drug safety signals from GLP‐1RA use, inverse associations with antidepressants and medications for SUD were observed, which are consistent with previous studies. These findings contribute to the growing and evolving body of real‐world evidence on the potential neuropsychiatric effects of GLP‐1RAs.

Our findings suggesting that GLP‐1RA use is inversely associated with subsequent initiation of antidepressants align with recent systematic reviews of RCTs and observational studies concluding that GLP‐1RAs may alleviate depressive symptoms and reduce the risk of depression.^23^, ^24^ Although the underlying pharmacological mechanism remains unclear, GLP‐1RA use has been shown to directly modulate pathways involved in mood regulation by activating GLP‐1R expressed in the hippocampus, which could indirectly modulate amygdala function.^25^, ^26^, ^27^ It is also possible that improvements in glycaemic control and diabetes‐related symptoms contribute to better mood.^28^ For example, diabetes‐related microvascular dysfunction has also been linked to a higher risk of depression.^29^ It is worthwhile mentioning that early pharmacovigilance and case studies have raised concern about GLP‐1RA use and suicidal ideation,^8^, ^9^ but several post‐marketing pharmacoepidemiologic studies have since found no association between GLP‐1RA use and suicidal risk.^30^, ^31^, ^32^

Evidence from this study indicating an inverse association between GLP‐1RA use and medications used for SUD contributes to the rapidly expanding body of clinical research on the potential protective effects of otherwise clinically indicated GLP‐1RAs against SUD, namely alcohol, tobacco, and opioid use disorders.^33^, ^34^ A recently published Phase II RCT demonstrated that semaglutide reduced alcohol craving and intake.^35^ A previous systematic review of RCTs and observational studies also concluded that there is growing evidence suggesting GLP‐1RA use may reduce alcohol consumption.^36^ As for tobacco use disorder, there is an RCT currently underway to evaluate dulaglutide as a new therapy for smoking cessation.^37^ Lastly, a large US observational study of people with existing opioid and alcohol use disorder reported that the use of GLP‐1RA was associated with lower rates of opioid overdose and alcohol intoxication.^38^ These clinical evidence is supported by preclinical models that suggest GLP‐1RA may influence reward processing pathways in the ventral tegmental area and nucleus accumbens, particularly through modulation of central dopamine signaling.^39^, ^40^

We found no associations between GLP‐1RA use and subsequent initiation of medications for neurodegenerative conditions such as Parkinson's and Alzheimer's disease. This contrasts with emerging clinical evidence from observational cohort studies suggesting that GLP‐1RA use may reduce the risk of these neurodegenerative conditions.^41^, ^42^ Preclinical studies have shown that GLP‐1RA can provide neuroprotective benefits through the modulation of inflammation, mitochondrial function, tau hyperphosphorylation, and anti‐apoptotic pathways.^43^ However, a systematic review and meta‐analysis of five Phase II RCTs reported inconclusive results on the effect of GLP‐1RA use in treating Parkinson's disease.^44^ A recently published Phase III RCT reported no evidence to support exenatide as a disease‐modifying treatment for Parkinson's disease.^45^ As for Alzheimer's disease, there are still two large Phase III RCTs underway to evaluate semaglutide as a disease‐modifying therapy for the early stage of the condition.^37^, ^46^ In this study, the lack of observed association should be interpreted alongside the relatively short follow‐up period, given the known long induction period for disease development and long latency due to diagnostic delays common in neurodegenerative conditions. Furthermore, there was a small number (*n*~100) of people initiating both GLP‐1RA and antidementia medications in our study, and not everyone with dementia will be prescribed antidementia medications.

In the sensitivity analyses using an extended two‐year exposure window, we observed inverse associations with subsequent initiation of medications for schizophrenia, epilepsy, and migraine. It is possible that these findings may indicate delayed or cumulative effects of GLP‐1RAs on these conditions. A meta‐analysis of 27 RCTs reported that GLP‐1RAs reduced the combined risk of seizures and epilepsy.^47^ However, these findings should be interpreted cautiously given the risk of time‐varying within‐person confounding over the longer exposure time window, e.g., aging, accumulating comorbidities, or medication(s) use. Moreover, the corresponding marker medications are also indicated for other conditions, for example, antiepileptics for bipolar disorder and migraine prophylaxis. A cohort study suggests that liraglutide may reduce headache frequency in patients with obesity and treatment‐resistant migraine, and that this effect appears independent of weight loss.^48^

These findings have significant implications for future research and safety monitoring of GLP‐1RAs. This PSSA approach is a crude signal detection method to identify potential drug safety and repurposing signals using real‐world data. Our results support the need for further targeted observational studies with tailored confounding control to confirm these neuropsychiatric effects, and further preclinical studies to understand the underlying biological and pharmacological mechanisms. Given the expanding use of GLP‐1RAs globally and here in Australia for new indications such as weight management,^4^, ^5^ regulatory agencies and researchers should continue evaluating the ongoing safety of GLP‐1RA use in post‐marketing surveillance efforts. Our findings also continue to support the possibility of repurposing GLP‐1RA for indications beyond glycaemic control and weight management. For now, a better understanding of these associations will be valuable to inform clinical decision‐making, for example, in tailored prescribing of glucose‐lowering medications in people with different baseline neurological or psychiatric risk profiles.

Our study has several strengths. We utilized a nationwide representative and longitudinal prescription claims database to capture individual‐level medication dispensing. We utilized the well‐established PSSA method, which was shown to have high specificity and moderate sensitivity to detect associations between index and marker medications.^19^ To maximize transparency and reproducibility of research, we have clearly outlined the specifications of our PSSA study design according to Hendrix *et al*.^20^ We have also conducted a range of sensitivity analyses by varying time windows in the study design to assess the robustness of our findings.

However, there are several important limitations. First, we are unable to delineate the effects of improved glycemic control when evaluating these associations, as GLP‐1RAs are only subsidized under the PBS for type 2 diabetes and thus everyone in our data received GLP‐1RA for management of type 2 diabetes. Second, although PSSA design controls for time‐invariant confounders, it is still susceptible to time‐varying within‐person confounding, e.g., change in health status or health behavior. Similar to most PSSA studies, we limited the exposure period to one‐year in the primary analyses to minimize the risk of time‐varying confounding. Third, the detection of spurious associations due to reverse causation is possible. For example, individuals undergoing smoking cessation therapies may experience weight gain, leading to initiation of GLP‐1RAs for weight management. Additionally, the use of antidepressants, particularly mirtazapine and tricyclic antidepressants, could increase the possibility of weight gain, and hence the use of GLP‐1RAs. Fourth, our prescription claims data did not capture private and over‐the‐counter medications. Private prescription of GLP‐1RAs and over‐the‐counter medication use, such as for acute migraines and nicotine dependence, were not considered in our analyses. Lastly, not everyone with these neuropsychiatric conditions is prescribed medications, and some marker medications may have been used for other indications, which may lead to misclassification of outcomes. Lastly, we did not adjust for multiplicity in this study, given its exploratory signal‐detection nature across a defined number of medications for neuropsychiatric conditions. Our goal was not to prioritize a long list of signals for further evaluation, such as when using multiplicity‐adjusted *P*‐values in tree‐based scan statistics,^49^ and other outcome‐wide approaches. However, the use of a more conservative confidence interval, e.g., 99% CI, may be considered when conducting hypothesis‐free PSSA across a much broader range of associations,^50^ but this will be at the expense of decreasing the likelihood of identifying true signals. Given this, the findings of our study should be interpreted in the context of multiple outcome groups, with repeated analytic windows.

## CONCLUSION

In conclusion, PSSA results demonstrated that GLP‐1RA use was inversely associated with subsequent initiation of antidepressants and medications for SUD. There was no clear association with other neuropsychiatric medications. While our findings partially align with existing clinical evidence, future preclinical and clinical studies are warranted to fully characterize the neuropsychiatric effects of GLP‐1RAs. Collectively, this evidence will inform safe and effective real‐world use of GLP‐1RAs and even drive investigations to repurpose GLP‐1RA for new indications.

## FUNDING

AJC is supported by a National Health and Medical Research Council Emerging Leadership 1 grant (APP2009633). DG is supported by PharmAlliance Clusters for Doctoral Training Scholarships. SW is supported by the Commonwealth through an Australian Government Research Training Program Scholarship [DOI: https://doi.org/10.82133/C42F‐K220].

## CONFLICTS OF INTEREST

J.I. reports grants from AstraZeneca, Amgen, National Health and Medical Research Council, Australian Government Department of Health, Disability and Aging, outside the submitted work. AJC has received grant funding or consulting funds from the National Health and Medical Research Council, Medical Research Future Fund, Dementia Australia Research Foundation, and Pharmaceutical Society of Australia. All grants and consulting funds were paid to the employing institution. All other authors declared no competing interests for this work.

## AUTHOR CONTRIBUTIONS

M.J.A.A., S.W., D.G., and G.S.Q.T. wrote the manuscript. M.J.A.A, S.W., D.G., J.I., A.J.C., M.L., and G.S.Q.T. designed the research. S.W. and G.S.Q.T. performed the research. M.J.A.A., S.W., D.G, J. I and G.S.Q.T. analyzed the data.

## Supporting information


Tables S1–S3

## Data Availability

The study data are not publicly available due to ethical and privacy constraints, but requests to the corresponding author for non‐individual‐level data will be considered on a case‐by‐case basis.
